# The Illustrated News

**Published:** 1881-08-20

**Authors:** 


*The Illustrated Scientific News*, published by Munn & Co., 37 Park Row, New York, is a most interesting and instructive paper.

